# Bloodstream infections and sepsis in Greece: over-time change of epidemiology and impact of de-escalation on final outcome

**DOI:** 10.1186/1471-2334-14-272

**Published:** 2014-05-18

**Authors:** Marina Koupetori, Theodoros Retsas, Nikolaos Antonakos, Glykeria Vlachogiannis, Ioannis Perdios, Christos Nathanail, Konstantinos Makaritsis, Antonios Papadopoulos, Dimitrios Sinapidis, Evangelos J Giamarellos-Bourboulis, Ioannis Pneumatikos, Charalambos Gogos, Apostolos Armaganidis, Elisabeth Paramythiotou

**Affiliations:** 1 1st Department of Internal Medicine, Thriassio General Hospital, Elefsis, Greece; 2 Department of Therapeutics, University of Athens, Medical School, Athens, Greece; 3 4th Department of Internal Medicine, University of Athens, Medical School, 1 Rimini Street, 12462 Athens, Greece; 4 Intensive Care Unit, Aghios Dimitrios General Hospital, Thessaloniki, Greece; 5 Intensive Care Unit, Artas General Hospital, Artas, Greece; 6 Department of Internal Medicine, General Hospital of the University of Thessaly, Larissa, Greece; 7 1st Department of Internal Medicine, G.Gennimatas General Hospital, Athens, Greece; 8 Intensive Care Unit, Democriteion University Hospital, Alexandroupolis, Greece; 9 Department of Internal Medicine, University of Patras, Rion, Greece; 10 2nd Department of Critical Care Medicine, University of Athens, Medical School, Athens, Greece

**Keywords:** Bloodstream infections, Sepsis, De-escalation, Resistance

## Abstract

**Background:**

Choice of empirically prescribed antimicrobials for sepsis management depends on epidemiological factors. The epidemiology of sepsis in Greece was studied in two large-periods.

**Methods:**

Sepsis due to bloodstream infections (BSI) from July 2006 until March 2013 was recorded in a multicenter study in 46 departments. Patients were divided into sepsis admitted in the emergencies and hospitalized in the general ward (GW) and sepsis developing after admission in the Intensive Care Unit (ICU). The primary endpoints were the changes of epidemiology and the factors related with BSIs by multidrug-resistant (MDR) pathogens; the secondary endpoint was the impact of de-escalation on antimicrobial therapy.

**Results:**

754 patients were studied; 378 from 2006–2009 and 376 from 2010–2013. Major differences were recorded between periods in the GW. They involved increase of: sepsis severity; the incidence of underlying diseases; the incidence of polymicrobial infections; the emergence of *Klebsiella pneumoniae* as a pathogen; and mortality. Factors independently related with BSI by MDR pathogens were chronic hemofiltration, intake of antibiotics the last three months and residence into long-term care facilities. De-escalation in BSIs by fully susceptible Gram-negatives did not affect final outcome. Similar epidemiological differences were not found in the ICU; MDR Gram-negatives predominated in both periods.

**Conclusions:**

The epidemiology of sepsis in Greece differs in the GW and in the ICU. De-escalation in the GW is a safe strategy.

## Background

Bloodstream infections (BSIs) are a significant cause of mortality especially if they are manifested with signs of sepsis and septic shock [1], [2]. Initial antimicrobial therapy, although empirical, has an important impact upon the survival of patients with sepsis and BSI [3-5]. Since microbiology results become available after 48 to 96 hours, appropriate selection of initially administered antimicrobials depends on factors of epidemiology suggesting both the most likely pathogen and the probable susceptibility profile. The same applies for patients developing sepsis and BSI after admission to an Intensive Care Unit (ICU). In this case, initial choice of antimicrobials is more difficult since multidrug-resistant (MDR) pathogens predominate in the nosocomial ICU environment.

Nowadays it is recognized that when initial coverage with broad-spectrum antimicrobials is administered to cover all probable pathogens and to improve outcomes, the chances to provide antimicrobial selection pressure ending with the predominance of MDR isolates are significantly increased [6]. One of the strategies recommended to avoid selection of MDR isolates is antibiotic de-escalation [7]. This strategy includes either switching to a narrower spectrum antimicrobial when the antibiogram is available or reducing the number of antibiotics used. The application of this strategy is nevertheless limited with a rate varying between 22 and 74% of cases particularly when this concerns patients with ventilator-associated pneumonia (VAP) [8]–[10].

Greece is traditionally regarded as an environment of high-selection pressure for the emergence of resistant isolates due to the over-consumption of antimicrobials both in the community and in the hospitals [11]. The changing epidemiology of sepsis in Greece may be used as a prototype for similar environments worldwide suffering by predominance of MDR pathogens. Selection of empirically administered antimicrobials in critically ill patients with sepsis relies on the knowledge of the following: a) over-time change of the epidemiology of implicated pathogens; b) epidemiological factors that can predict implication of MDR pathogens; and c) the outcome of de-escalation of antimicrobials. To answer these questions, a prospective multicenter study was conducted in Greece from 2006 until 2013.

## Methods

### Study design

This is an ongoing prospective observational study conducted since September 2006 in 46 departments coming from 31 hospitals in Greece. From these departments, 21 are mixed surgical/medical ICUs; 20 are departments of internal medicine; and five are departments of general surgery. The protocol was approved by the Ethics Committee of each hospital. Written informed consent was collected from all patients or from first-degree relatives for patients unable to consent. Patients with community-acquired sepsis admitted to the general ward or with hospital-acquired sepsis developing in the ICU at least 48 hours after ICU admission are enrolled in this study. Participating departments were not obliged to report all cases of sepsis admitted since the beginning of the study. Data referring to patients with BSIs due to sepsis collected until March 2013 were analyzed in the current manuscript.

Inclusion criteria were: a) age ≥18 years; b) at least two signs of the systemic inflammatory response syndrome; and c) presence of BSI. Exclusion criteria were: a) infection by the human immunodeficiency virus; b) neutropenia defined as less than 1000 neutrophils/mm^3^ due to causes other than sepsis; c) death of patients before the results of blood cultures were available.

The following signs of SIRS were considered for study enrollment [12]: a) core temperature >38°C or <36°C, b) p_co2_ < 32 mmHg or more than 20 breaths/min, c) pulse rate >90/min, and d) white blood cells >12,000/mm^3^ or <4,000/mm^3^ or >10% of band forms. Patients were classified as suffering from sepsis, severe sepsis and septic shock according to standard definitions [12].

BSI was considered as the isolation of at least one Gram-negative or Gram-positive or one fungal species from blood sampled from a peripheral vein. If coagulase-negative *Staphylococcus* spp (*CoNS*) and other common skin flora were isolated, they were considered as skin contaminants if they were isolated in a single blood culture. When *CoNS* was isolated both from a central and a peripheral vein with the same antibiogram, it was considered true pathogen.

When no underlying infection was diagnosed despite intense clinical and radiological work-out, BSI was considered primary. In all other cases it was considered secondary to another infection. For the cases of secondary BSIs, diagnosis of acute pyelonephritis, community-acquired pneumonia, intraabdominal infections, ventilator-associated pneumonia and acute bacterial skin and soft tissues infections were done by standard definitions [13]–[15].

Only one septic episode for each patient was studied. Polymicrobial BSI was identified when at least two different microorganisms were isolated from blood within a 48-h period. The following information were recorded: demographics; Acute Physiology and Chronic Health Evaluation (APACHE) II score; laboratory examinations; source of BSI; administered antimicrobial agents; isolated pathogens from the bloodstream and their antibiogram; co-existing diseases; predisposing conditions; and 28-day outcome expressed as crude mortality. Prior exposure to an antimicrobial agent was elaborated both by the patients’ case history and by monitoring of the prescribed drugs over the last three months.

For blood culturing, 10 ml of blood was inoculated into ready-prepared culture vials (bioMérieux, Marcy l’Etoile, France) and incubated into the Vitek 2 automated system. The same system reported for identification of microorganisms and for susceptibility testing by measurement of the minimum inhibitory concentrations (MICs) of antimicrobials onto pre-coated microplates. Susceptibility was reported based on the CLSI susceptibility criteria. For species of *Staphylococcus aureus* susceptibility to methicillin was defined by the inhibition of growth around a disk of 30 μg of cefoxitin after plating onto Muller-Hinton agar. Susceptibility of Gram-negative species to colistin was assessed by a similar way using a 10 μg disk.

Administered antimicrobial therapy was considered appropriate when the isolated microorganism was susceptible to at least one of the administered antimicrobials. Any bloodstream isolate resistant to at least three drugs of different classes was considered multidrug-resistant (MDR).

### Study end-points

The primary study endpoints were the changes of epidemiology of BSIs causing sepsis over the 7-year time period and the factors related with BSIs by MDR pathogens.

The secondary study endpoint was the impact of de-escalation of antimicrobial therapy on the final outcome.

### Statistical analysis

Episodes of BSIs were studied into two equal study time periods; from September 2006 until December 2009 and from January 2010 until April 2013. In each study period, patients were divided into two groups: those admitted with sepsis in the emergency department and who were hospitalized in the general ward (GW) and into those who developed sepsis at least 48 hours after ICU admission. Comparisons between groups were done by the X^2^ test for qualitative variables and by the Student’s “t-test” for the quantitative variables. In order to assess factors related with BSI by one MDR pathogen, univariate analysis was done only for the GW group. Those factors with a p value of significance lower than 0.100 entered as independent variables into a logistic regression analysis model. Odds ratios (ORs) and 95% confidence intervals (CI) were determined.

Only patients in the GW with BSIs by fully susceptible Gram-negative isolates of both study periods were analyzed for the secondary endpoint. As such de-escalation therapy was considered as a switch to a drug class covering a narrower spectrum of microorganisms. The decision for de-escalation was made according to the discretion of the attending physicians. Patients of each study period were divided into the de-escalation arm and into the non-de-escalation arm. Survival of each arm was measured by Kaplan-Meier analysis; comparisons were done by the log-rank test. Since the study was observational, to assess the impact of sepsis severity on final outcome, forward Cox-logistic regression analysis was done; presence of severe sepsis/shock, age, gender, history of at least one underlying disease and de-escalation were entered as independent variables in the equation. Hazard ratios (HRs) and 95% CIs were determined. Any p value below 0.05 was considered significant.

## Results

### Study flow

**Figure 1:**
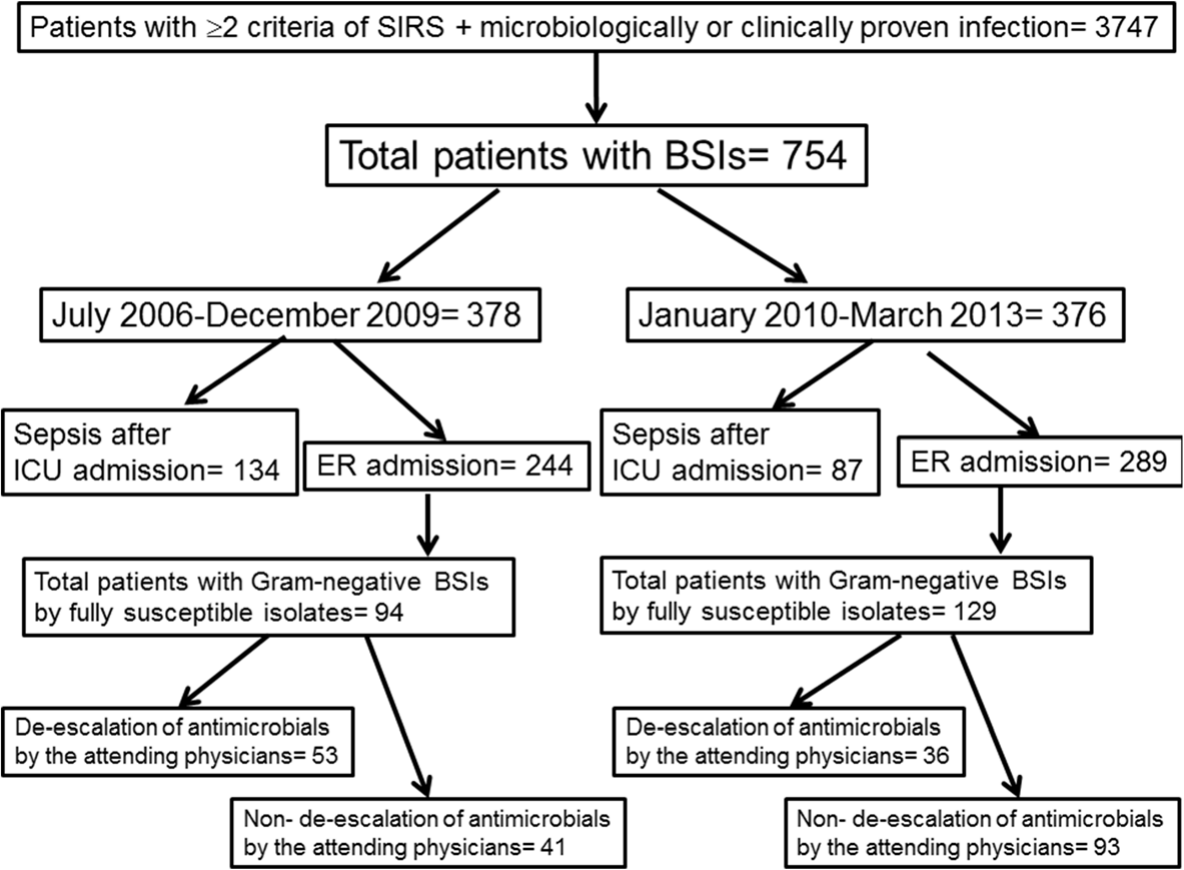
**Study flow-chart.** Abbreviations BSI: bloodstream infection; ER: emergency department; ICU intensive care unit; SIRS: systemic inflammatory response syndrome.

Blood sampling was done prior to administration of the first dose of empirical antimicrobial therapy in 773 out of 1814 patients (42.9%) screened during the 2006–2009 study period and in 748 out of 1292 patients (57.9%) screened during the 2010–2013 study period (p < 0.0001 between the two study periods). The study flow-chart is shown in Figure 1. According to this, a total of 754 cases of BSIs with sepsis were studied for the primary study endpoint; 378 BSIs from the 2006–2009 study period; and 376 BSIs from the 2010–2013 study period; 94 and 129 BSIs respectively met the criteria to be studied for the impact of de-escalation of antimicrobials on the final outcome.

### Primary end-point

The primary study end-point was the changes of the characteristics of severe BSIs during the two study periods. Demographic and clinical characteristics shown in Table 1 indicate the absence of any differences between the populations hospitalized in the ICUs. On the contrary, major differences were recorded between the two study periods for patients hospitalized in the GW. These changes involved mainly an increase of sepsis severity and of the incidence of underlying diseases from the first to the second study period but also a reciprocal increase of mortality. Logistic regression analysis of these changes revealed that increase of type 2 diabetes mellitus was the main difference between the two study periods (Table 2).

**Table 1:** Demographic and clinical characteristics of patients with sepsis and bloodstream infections of the two study periods

	**2006**-**2009**	**2010**-**2013**	**p**
**Admission in the emergencies and general ward hospitalization**			
Male gender (n,%)	120 (49.8)	121 (42.6)	0.114
Age (years, mean ± SD)	70.2 ± 16.6	73.3 ± 15.3	0.034
APACHE II (mean ± SD)	14.33 ± 8.57	17.87 ± 8.27	<0.0001
White blood cell count (/mm^3^, mean ± SD)	15801.5 ± 11733.0	16680.9 ± 10705.6	0.371
Sepsis severity (n,%)			
Uncomplicated sepsis	152 (62.3)	151 (52.2)	0.022
Severe sepsis/shock	92 (37.7)	138 (47.8)	
Underlying type of infection (n,%)			
Acute pyelonephritis	119 (48.8)	148 (51.2)	
Community-acquired pneumonia	19 (7.8)	22 (7.7)	
Intrabdominal infections	32 (13.1)	38 (13.1)	0.052
Primary bacteremia	69 (28.3)	71 (24.6)	
Soft-tissue infections	3 (1.2)	4 (1.4)	
Catheter-related bacteremia	2 (0.8)	6 (2.1)	
Underlying diseases (n,%)			
Type 2 diabetes mellitus	61 (25.0)	118 (40.8)	<0.0001
Chronic heart failure	42 (17.2)	70 (24.2)	0.055
COPD	13 (5.3)	27 (9.3)	0.099
Chronic renal disease	15 (6.1)	32 (11.1)	0.048
Systemic oral corticosteroids	7 (2.9)	15 (5.2)	0.197
Solid tumor malignancy	49 (20.1)	46 (15.9)	0.214
Predisposing conditions (n,%)			
Ischemic stroke	33 (13.5)	61 (21.1)	0.023
Dementia	26 (10.7)	20 (6.9)	0.163
Gallstones	29 (11.9)	22 (7.6)	0.105
Renal stones	36 (14.8)	49 (17.0)	0.553
Appropriateness of administered antibiotics (n,%)	227 (93.0)	233 (91.4)	0.364
Mortality (n,%)	45 (18.4)	95 (32.9)	<0.0001
**Sepsis developing after ICU admission**			
Male gender (n,%)	83 (66.9)	51 (61.4)	0.459
Age (years, mean ± SD)	63.6 ± 15.9	61.9 ± 19.67	0.489
APACHE II (mean ± SD)	21.57 ± 7.15	20.99 ± 8.16	0.589
White blood cell count (/mm^3^, mean ± SD)	14747.3 ± 10326.3	13193.4 ± 11167.0	0.293
Sepsis severity (n,%)			
Uncomplicated sepsis	33 (24.6)	19 (21.8)	0.634
Severe sepsis/shock	101 (25.4)	58 (78.2)	
Underlying type of infection (n,%)			
Ventilator-associated pneumonia	36 (26.9)	29 (33.3)	
Intrabdominal infections	1 (0.7)	3 (3.4)	0.034
Primary bacteremia	90 (67.2)	50 (57.5)	
Catheter-related bacteremia	7 (5.2)	5 (5.7)	
Underlying diseases (n,%)			
Type 2 diabetes mellitus	38 (28.4)	20 (30.0)	0.435
Chronic heart failure	40 (29.9)	21 (24.1)	0.441
COPD	41 (30.6)	17 (19.5)	0.085
Chronic renal disease	14 (10.4)	9 (10.3)	1.000
Systemic oral corticosteroids	9 (6.7)	13 (14.9)	0.064
Solid tumor malignancy	14 (10.4)	6 (6.9)	0.474
Predisposing condition (n,%)			
Ischemic stroke	30 (22.4)	17 (24.6)	0.728
Multiple injuries	10 (7.5)	10 (14.5)	0.137
Appropriateness of administered antimicrobials (n,%)	104 (77.6)	62 (71.7)	0.538
Mortality (n,%)	50 (37.3)	40 (45.9)	0.208

Data are expressed as the number of cases (%), except where specified.

*Abbreviations*: *COPD* chronic obstructive pulmonary disorder, *ICU* intensive care unit.

**Table 2:** Logistic regression analysis of factors related with differences of demographic and clinical characteristics of patients admitted with sepsis in the emergencies and hospitalized in the general ward between the two study periods

	**OR**	**95% CI**	**p**
Age	1.01	0.99-1.017	0.138
Type of infection	0.97	0.92-1.05	0.663
Presence of severe sepsis/shock	1.16	0.87-1.54	0.318
Type 2 diabetes mellitus	1.63	1.17-2.27	0.004
Chronic renal disease	0.98	0.57-1.69	0.948
Ischemic stroke	1.32	0.89-1.94	0.166

*Abbreviations*: *CI* confidence intervals, *OR* odds ratio.

**Figure 2:**
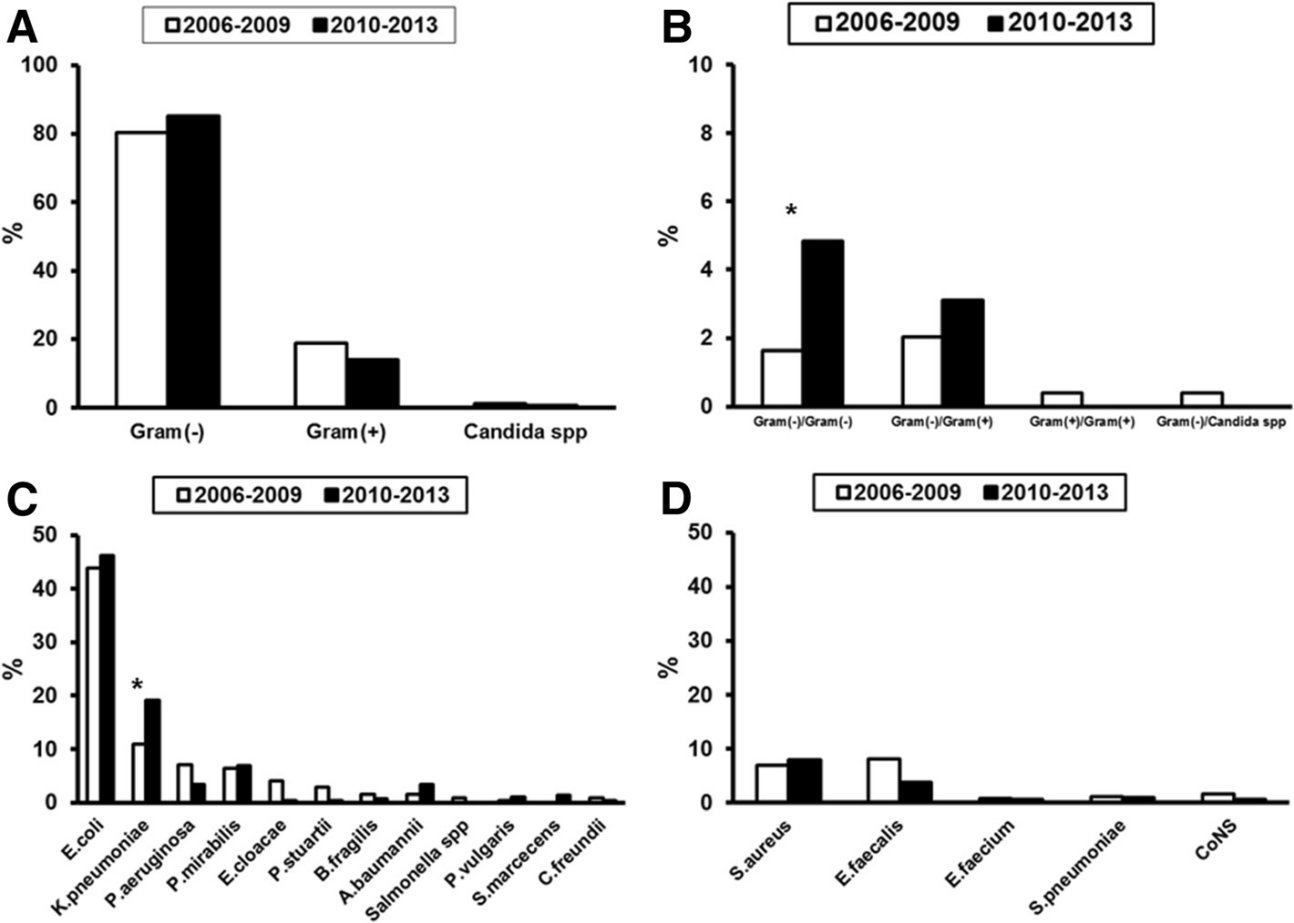
**Distribution of bloodstream pathogens between the two study periods for patients admitted with sepsis in the emergencies and hospitalized outside the ICU. A)** Pathogens are distributed according to Gram stain or fungal species; **B)** Mixed infections are presented; **C)** Distribution of species of Gram (−) bacteria; and **D)** Distribution of species of Gram (+) cocci. Asterisks indicate statistical significances between the two study periods. CoNS: coagulase negative *Staphylococcus* spp.

The distribution of pathogens for patients hospitalized outside the ICU for sepsis is shown in Figure 2. Polymicrobial infections of both Gram-positive and Gram-negative etiology increased from the first study period to the second study period. A similar increase has also been shown for infections caused by *Klebsiella pneumoniae*.

**Figure 3:**
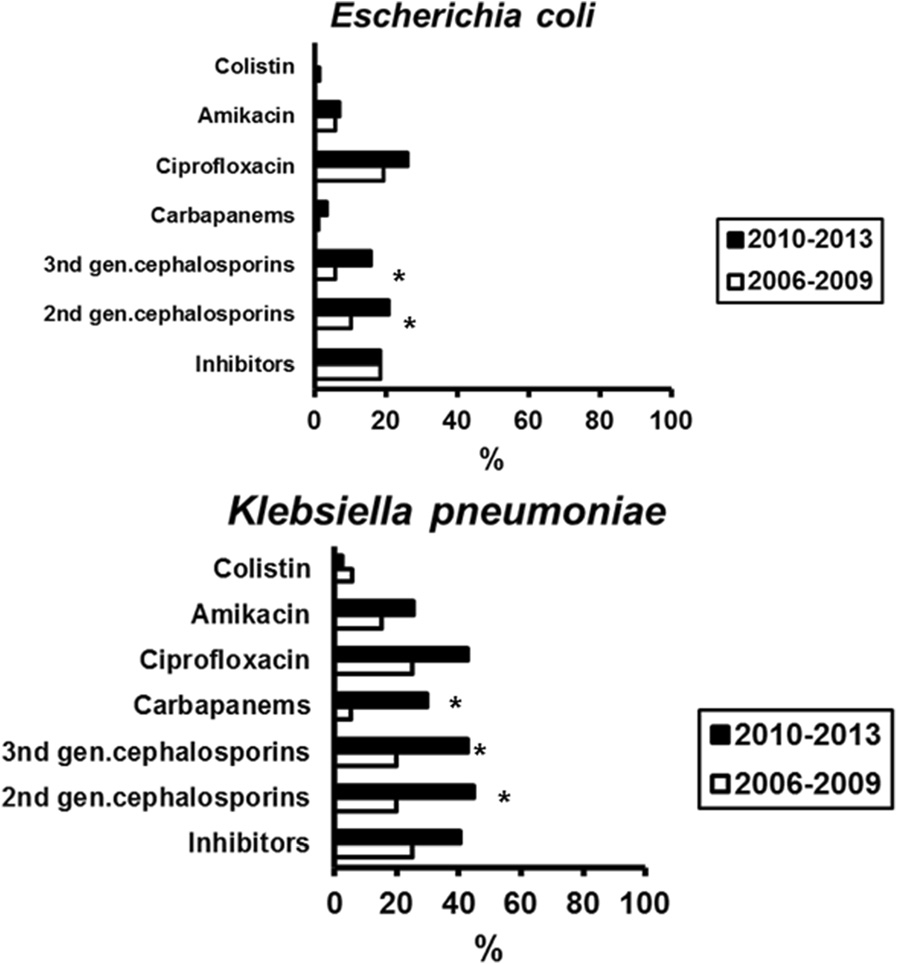
**Resistance rates of isolates of *Escherichia coli *and of *Klebsiella pneumoniae *of patients admitted with sepsis and hospitalized outside the ICU for the two study periods.** Asterisks indicate statistical significances between the two study periods.

This change in pathogen distribution was accompanied by changes in resistance rates. The most frequent isolates were *E.coli* and *K.pneumoniae*. Their resistance patterns are shown in Figure 3. Regarding *E.coli*, resistance rates for 2nd and 3rd generation cephalosporins were increased from the 2006–2009 period to the 2010–2013 period. Similar increases were described for *K.pneumoniae* involving the great majority of the studied antimicrobials. Production of extended-spectrum β-lactamases (ESBL) was found in 16.4% of Gram-negative isolates of the 2006–2009 study period and in 27.1% of Gram-negative isolates of the 2010–2013 study period (p: 0.016). Production of KPC carbapenemases was found in 7.5% and 14.8% of Gram-negative isolates respectively (p: 0.031). Resistance of *S.aureus* to methicillin was 25% for the 2006–2009 isolates and 37.5% for the 2010–2013 isolates (p: 0.447).

These findings generated the question which factors from the patients’ history are related to a greatest risk for infection by MDR pathogens among patients in the GW. Since patients of the two study periods differed in their demographic characteristics, patients of both periods were encountered together. To this end, univariate analysis indicated for a positive relationship with APACHE II score more than 13 (p: 0.083); history of chronic obstructive pulmonary disease (p: 0.083); presence of a pig-tail ureteral catheter (p: 0.084); chronic hemofiltration (p: 0.001); intake of antibiotics the last three months (p: 0.056); and residence into a long-term care facility (LTCF) (p: 0.011). Logistic regression analysis was then performed taking into consideration all these factors as independent variables in the equation (Table 3). This analysis indicated that the only factors independently related with BSI by MDR pathogens were chronic hemofiltration, intake of antibiotics the last three months and residence into one LTCF.

**Table 3:** **Logistic regression analysis of factors related with the occurrence of infections by multidrug**-**resistant pathogens of patients admitted with sepsis in the emergencies and hospitalized in the general ward**

	**OR**	**95% CI**	**p**
APACHE II > 13	1.57	0.79-3.09	0.192
History of COPD	2.61	0.78-8.77	0.120
Pigtail ureter catheterization	4.67	0.94-23.23	0.060
Chronic hemofiltration	7.16	1.93-26.54	0.004
Intake of antibiotics the last 3 months	2.48	1.34-4.57	0.004
Residence in one LTCF	4.62	2.12-10.10	<0.0001

*Abbreviations*: *APACHE* acute physiology and chronic health evaluation score, *CI* confidence intervals, *COPD* chronic obstructive pulmonary disorder, *LTCF* long-term care facility, *OR* odds ratio.

**Figure 4:**
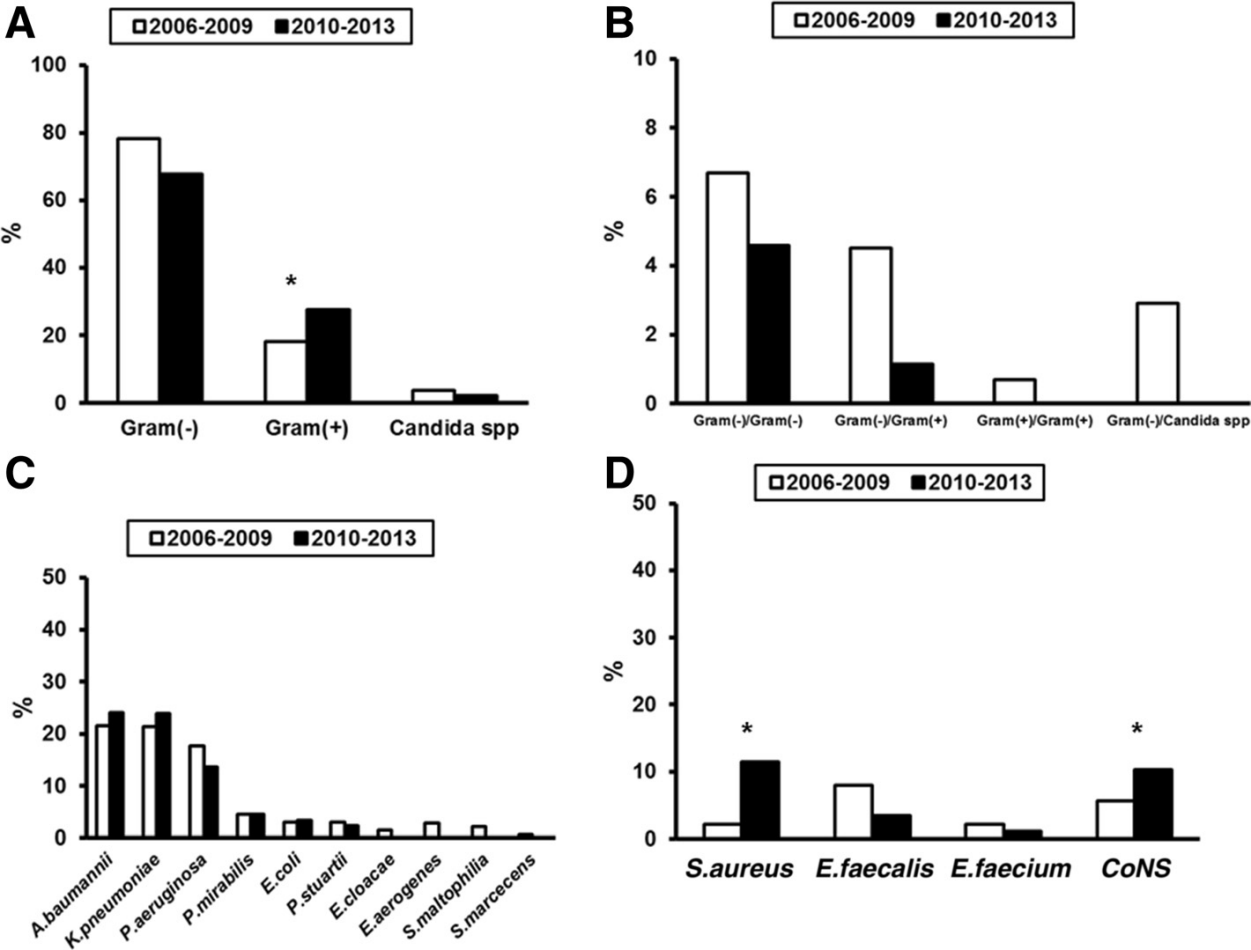
**Distribution of bloodstream pathogens between the two study periods for patients with sepsis developing after ICU admission. A)** Pathogens are distributed according to Gram stain or fungal species; **B)** Mixed infections are presented; **C)** Distribution of species of Gram (−) bacteria; and **D)** Distribution of species of Gram (+) cocci. Asterisks indicate statistical significances between the two study periods. CoNS: coagulase negative *Staphylococcus* spp.

**Figure 5:**
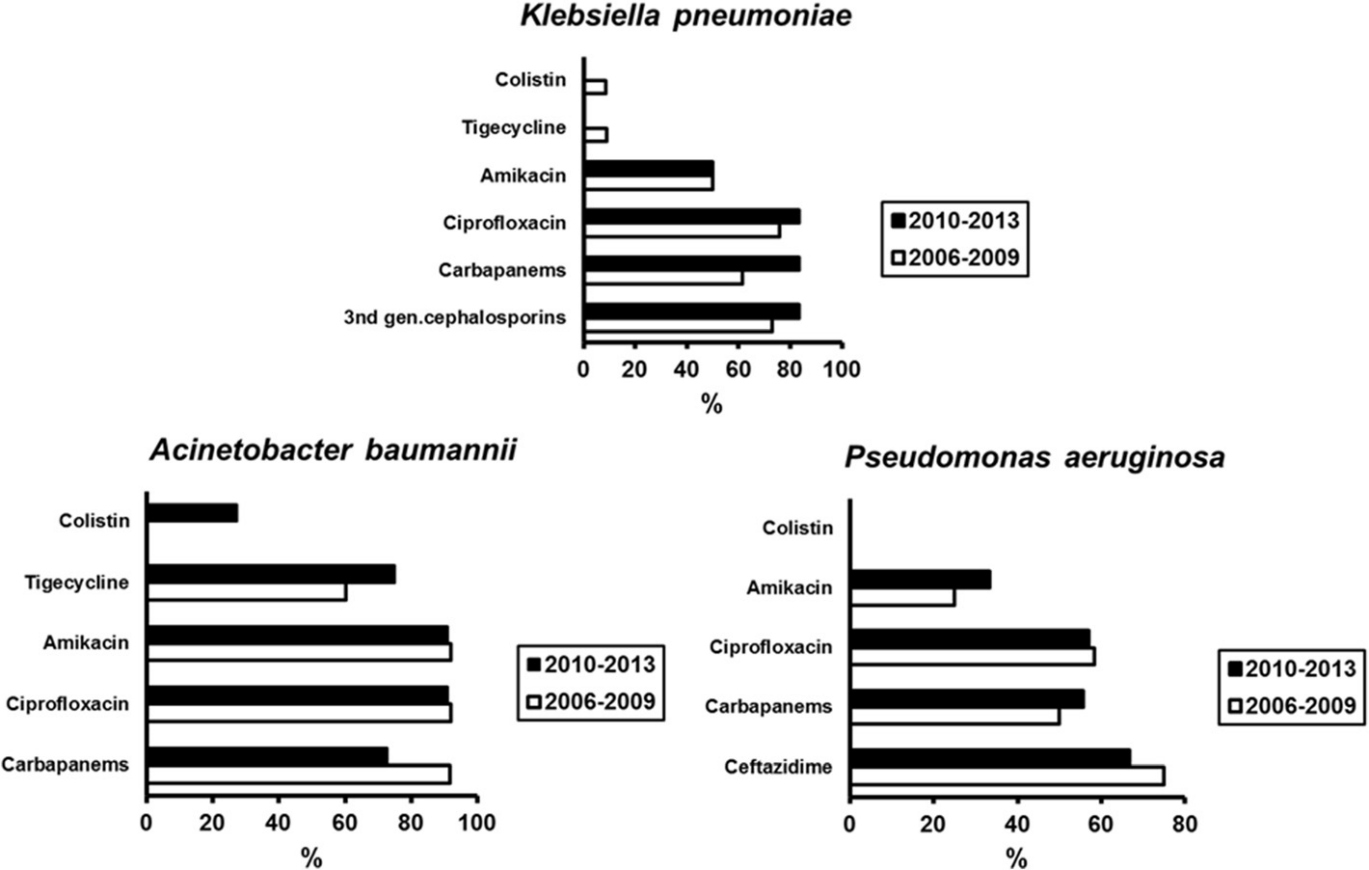
**Resistance rates of isolates of *Klebsiella pneumoniae***, **of *Acinetobacter baumannii *and of *Pseudomonas aeruginosa *of patients developing sepsis after ICU admission of the two study periods.**

The distribution of pathogens for patients developing sepsis after ICU admission is shown in Figure 4. As it can be seen, infections by *S.aureus* and by *CoNS* increased from the 2006–2009 period to the 2010–2013 study periods. Resistance of these species to methicillin was 95.9% for the 2006–2009 isolates and 100% for the 2010–2013 isolates (p: 1.000). Resistance rates for *K.pneumoniae*, *A.baumannii* and *P.aeruginosa* that were the main Gram-negative pathogens did not change between the two study periods (Figure 5). Production of ESBL was found in 76.6% of Gram-negative isolates of the 2006–2009 study period and in 71.1% of Gram-negative isolates of the 2010–2013 study period (p: 0.371). Production of KPC carbapenemases was found in 60.9% and 64.9% of Gram-negative isolates respectively (p: 0.431).

### Secondary end-point

**Figure 6:**
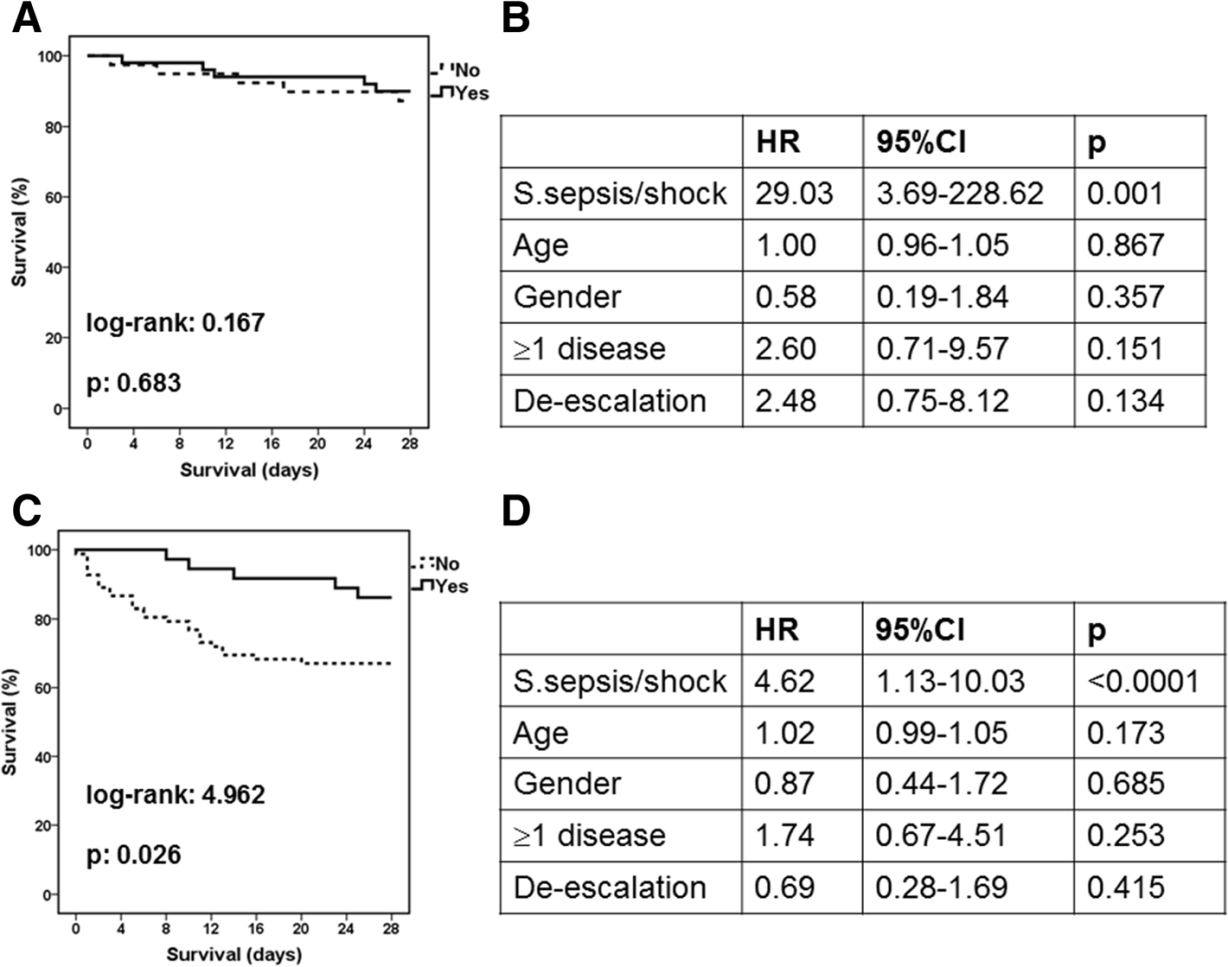
**Impact of de**-**escalation on final outcome.** Kaplan Meier survival analysis was conducted comparing patients for which antimicrobials were de-escalated by the attending physicians (Yes, solid line) with those for which antimicrobials were not de-escalated by the attending physicians (No, dashed line) separately for the study period 2006–2009 (panel **A)** and for the study period 2010–2013 (panel **C)**. P values refer to comparisons between patients under de-escalation and patients without de-escalation. Panels **B** (study period 2006–2009) and **D** (study period 2010–2013) refer to forward Cox regression analysis of the impact of de-escalation on the final outcome after adjustment for the presence of severe sepsis/shock, age, gender and for comorbid illnesses. CI: confidence intervals; HR: hazard ratio.

From the patients enrolled during the first study period, 94 were hospitalized in the GW with BSI by fully susceptible Gram-negative pathogens; antimicrobials were de-escalated in 53 patients and non-de-escalated in 41 patients. From the patients enrolled during the second study period, 129 patients were hospitalized in the GW with BSI by fully susceptible Gram-negative pathogens; antimicrobials were de-escalated in 36 patients and non-de-escalated in 93 patients. Mean ± SD APACHE II of patients not subjected to de-escalation of antimicrobials during the period 2006–2009 was 14.56 ± 8.63; it was 13.50 ± 11.51 for patients subjected to de-escalation of antimicrobials of the same study period (p: 0.632). Respective values for the study period 2010–2013 were 18.18 ± 7.28 and 13.04 ± 5.89 (p < 0.0001). Kaplan-Meier analysis showed no statistical difference in final outcome between patients subjected and not subjected to de-escalation of antimicrobials of the first study period (Figure 6A). Prolonged survival was found for patients subjected to de-escalation compared to patients not-subjected to de-escalation of antimicrobials during the second study period (Figure 6C). However the differences in APACHE II scores for the second study period, led to Cox regression analysis after adjustment for disease severity. Still, no impact of de-escalation on final outcome was found in any of the two study periods (Figure 6B and Figure 6D).

The type of administered antimicrobials after the antibiogram became known is shown in Table 4 being different between patients subjected to de-escalation and not subjected to de-escalation in both study periods.

**Table 4:** **Antimicrobial treatment in relation to de**-**escalation policy**

**Antimicrobial combination**	**Non**-**de**-**escalation**	**De**-**escalation**	**p**
	**2006**-**2009 study period(n,%)**		
β-lactamase inhibitors	0 (0)	15 (28.3)	
2nd generation cephalosporins	0 (0)	16 (30.2)	
3rd generation cephalosporins	6 (14.6)	2 (3.8)	
Piperacillin/tazobactam	27 (65.8)	3 (5.6)	<0.0001
Carbapanem	3 (.3)	0 (0)	
Carbapanem + glycopeptide	5 (12.2)	0 (0)	
Ciprofloxacin	0 (0)	17 (32.1)	
	**2010**-**2013 study period (n,%)**		
β-lactamase inhibitors	0 (0)	8 (22.2)	
2nd generation cephalosporins	0 (0)	15 (41.7)	
3rd generation cephalosporins	7 (7.5)	5 (13.9)	
Piperacillin/tazobactam	59 (63.4)	2 (5.5)	<0.0001
Piperacillin/tazobactam + glycopeptide	9 (9.7)	0 (0)	
Carbapanem	10 (10.8)	0 (0)	
Carbapanem + glycopeptide	8 (8.6)	0 (0)	
Ciprofloxacin	0 (0)	6 (16.7)	

Antimicrobials prescribed to patients after culture results and the antibiogram became known to the attending physicians are shown.

## Discussion

This study describes the trends of epidemiology of sepsis arising in the field of BSI both for patients admitted with sepsis from the ED and hospitalized in the GW and for patients developing sepsis after ICU admission over a 7-year period in Greece. The main findings are: the need to hospitalize more severe patients in the GW over the second study period as reflected by the higher prevalence of comorbidities and by the increased mortality; the increase of *K.pneumoniae* as a pathogen and of the resistance rates of Gram-negative pathogens; the increase of Gram-positive cocci as pathogens in the ICU; and the safe application of de-escalation policy.

This is the first multicenter study, to our knowledge, that provides clinical and microbiological characteristics of well-documented cases with BSI in two different time periods comprising patients coming from the community and from the ICU. The seven-year period of follow-up is divided into two equal periods of three years each i.e. 2006 to 2009 and 2010 to 2013. Similar information is not provided in any other published manuscript because published evidence does not come as early as in 2013. Much of the explanation for the differences between the two study periods relies on the greater frequency of type 2 diabetes mellitus which is a well-recognized risk factor for severe bloodstream infections [16]. The shortage for ICU beds in Greece may also be part of this explanation.

The pathogens responsible for community-acquired BSIs vary widely between countries [17]. In our study, Gram-negative microorganisms are the predominant pathogens for patients with sepsis admitted in the ED in both periods. Our findings agree with other studies demonstrating that in these settings *E.coli* is the most common cause of BSI [16], [18]; however *E.coli* pathogens of the second study period showed increased resistance to 2nd- and 3rd-generation cephalosporins. Moreover, the incidence of *K.pneumoniae* was considerably increased from the first to the second time period. This increase parallels the increase in resistance of this pathogen to cephalosporins, quinolones and carbapenems and confirms the fact that the presence of antimicrobial resistant bacteria increases the total burden of disease [19]. This trend in resistance is alarming considering the known phenomenon of the presence of *Klebsiella*- producing carbapenemase (KPC) in Greek hospitals [20]. The significant increase in resistance pattern of both *E.coli* and *K.pneumoniae* pathogens probably reflects the rise of ESBL in the community [18] and the ability of these two species of Enterobacteriacae to transmit their resistance genes through plasmids [21].

Early administration of antimicrobials active on the implicated pathogens is the key to appropriate management. The current study provides salient information about the choice of antimicrobials. Information coming from the patients’ history about recent contact with a health-care-associated setting like the conduct of chronic hemofiltration and residence in a long-term care facility and the recent intake of antimicrobials should guide initial treatment towards broad-spectrum antimicrobial coverage. The current study also supports the use of a de-escalation policy for sepsis caused by fully susceptible Gram-negative pathogens. De-escalation to narrower spectrum antimicrobials does not impact final outcome. It may even be suggested that this policy led to survival benefit in the second study period. However, the proportion of patients de-escalated in the second study period was decreased compared with the first study period. The study was not interventional for the implementation of the de-escalation but this was a choice of the attending physicians. It may be hypothesized that the decrease of proportion of de-escalated patients may be linked with a feeling of physicians that antimicrobial resistance increase over-time and a subsequent fear for the de-escalation strategy. This is one of few studies on the impact of de-escalation policy in sepsis. In a recent publication, Garnacho-Montero et al. [22] followed-up prospectively 712 admissions due to severe sepsis in an ICU; de-escalation performed in 219 patients was a protective factor against both 30-day and 90-day mortality.

The situation of Greek ICUs for patients with sepsis developing after ICU admission are similar to other European countries regarding demographics and mortality [19], [23]. However it should be underscored that MDR Gram-negative species of *K.pneumoniae*, *A.baumannii* and *P.aeruginosa* predominate although a significant increase of BSIs by Gram-positive cocci mainly of species of *S.aureus* and of *CoNS* is recorded between the two study periods. These findings suggest that initial empirical therapy of sepsis developing after ICU admission should contain anti-Gram-positive coverage and that selection of anti-Gram-negative coverage should rely on the local resistance surveillance.

## Conclusion

The findings of the current study suggest that the epidemiology of sepsis in Greece is different for patients admitted in the ED and for patients who develop sepsis after ICU admission. Although Gram-negative bacterial predominate in both settings, resistance profiles are quite different and they are driven by the history of antimicrobial consumption and the exposure to health-care-associated settings. Application of de-escalation is a safe strategy when susceptible pathogens are involved.

## Abbreviations

APACHE: Acute physiology and chronic health evaluation; BSI: Bloodstream infections; ESBL: Extended-spectrum β-lactamases; GW: General ward; KPC: *Klebsiella*- producing carbapenemase; MDR: Multidrug-resistant; SIRS: Systemic inflammatory response syndrome; VAP: Ventilator-associated pneumonia.

## Authors’ contribution

MK collected clinical data, analyzed data, wrote the manuscript and gave approval for the final version. TR, NA, GV, IP, CN, KM, AP, DS collected clinical data, analyzed data, drafted the manuscript and gave approval for the final version. EJGB, IP, CG, AA and EP analyzed data, drafted the manuscript and gave approval for the final version.

## Competing interests

None of the authors has any competing interest related with this submission.

## Pre-publication history

The pre-publication history for this paper can be accessed here:

http://www.biomedcentral.com/1471-2334/14/272/prepub
